# Identification of Molecular Subtypes and Key Genes of Atherosclerosis Through Gene Expression Profiles

**DOI:** 10.3389/fmolb.2021.628546

**Published:** 2021-04-28

**Authors:** Yujia Yang, Yue Cai, Yuan Zhang, Xu Yi, Zhiqiang Xu

**Affiliations:** ^1^Department of Neurology and Centre for Clinical Neuroscience, Daping Hospital, Army Medical University (Third Military Medical University), Chongqing, China; ^2^Department of Cardiology, Xijing Hospital, Fourth Military Medical University, Xi’an, China

**Keywords:** atherosclerosis, weighted gene co-expression analysis, immune cell infiltration, LASSO, SVM-RFE

## Abstract

Atherosclerotic cardiovascular disease (ASCVD) caused by atherosclerosis (AS) is one of the highest causes of mortality worldwide. Although there have been many studies on AS, its etiology remains unclear. In order to carry out molecular characterization of different types of AS, we retrieved two datasets composed of 151 AS samples and 32 normal samples from the Gene Expression Omnibus database. Using the non-negative matrix factorization (NMF) algorithm, we successfully divided the 151 AS samples into two subgroups. We then compared the molecular characteristics between the two groups using weighted gene co-expression analysis (WGCNA) and identified six key modules associated with the two subgroups. Kyoto Encyclopedia of Genes and Genomes (KEGG) and gene ontology (GO) enrichment analysis were used to identify the potential functions and pathways associated with the modules. In addition, we used the cytoscape software to construct and visualize protein–protein networks so as to identify key genes in the modules of interest. Three hub genes including *PTGER3*, *GNAI1*, and *IGFBP5* were further screened using the least absolute shrinkage and selection operator (LASSO) and support vector machine-recursive feature elimination (SVM-RFE) algorithms. Since the modules were associated with immune pathways, we performed immune cell infiltration analysis. We discovered a significant difference in the level of immune cell infiltration by naïve B cells, CD8 T cells, T regulatory cells (Tregs), resting NK cells, Monocytes, Macrophages M0, Macrophages M1, and Macrophages M2 between the two subgroups. In addition, we observed the three hub genes were positively correlated with Tregs but negatively correlated with Macrophages M0. We also found that the three key genes are differentially expressed between normal and diseased tissue, as well as in the different subgroups. Receiver operating characteristic (ROC) results showed a good performance in the validation dataset. These results may provide novel insight into cellular and molecular characteristics of AS and potential markers for diagnosis and targeted therapy.

## Introduction

Atherosclerosis (AS) is a chronic inflammatory disease in which atherosclerotic plaques are deposited on the walls of blood vessels and induce arterial stenosis. In general, atherosclerosis is usually asymptomatic in the early stages. However, increased severity of the disease causes various diseases, such as coronary artery disease, peripheral artery disease, and cerebrovascular disease (Kong et al., 2014; Tern et al., 2018). It has been reported that there was sharp increase in the incidence of AS in 2017, with the AS-related mortality rising to 31% (Stefanadis et al., 2017). In addition, the AS is characterized by vascular inflammation, endothelial dysfunction, plaque formation, and diminished oxygen supply to target organs (Jeong, 2010). Currently, the underlying molecular mechanism of AS is still unclear, and an understanding of the molecular pathogenesis of atherosclerosis process could contribute to develop individualized treatment strategies for AS patients.

Until now, most studies on AS were mainly focused on the cardiovascular and cerebrovascular diseases, except for lipid deposition theory and inflammatory response involving immune cell, such as monocytes/macrophages, neutrophils, and T cells involved in AS (Niccoli et al., 2018). Presently, the conventional approaches for treating AS involve regulating some risk factors including smoking, alcohol, genetic factors, hypertension, and hyperlipidemia to prevent the development of AS. After the development of AS, the treatment strategy switches to use of anti-platelet aggregation and hypolipidemic drugs. In addition, local vascular stents or bypass measures can be used for the management of AS. However, these therapeutic strategies have not been effective in the management of AS as seen by the high morbidity and mortality associated with AS (Kelly et al., 2018). Thus, there is urgent need to delineate the molecular pathogenesis of AS, identify potential therapeutic markers, and develop effective drugs. Moreover, the C-reactive protein (CRP) has been involved in multiple processes of AS and is the optimal inflammatory biomarker to the prognosis of atherosclerotic events (Kampoli et al., 2009). The fibrinogen, apolipoproteins, and interleukins are tightly correlated with the progression of AS and have a strong relevance in risk prediction (Montagnana et al., 2008). However, the clinical predictive value of these markers is poor (Revkin et al., 2007). Therefore, a reliable and specific biomarker for promoting the clinical diagnosis of atherosclerosis is urgently required.

The present study aimed to identify potential subgroups of AS through integrating multiple datasets based on the gene expression profile. The protein–protein interaction (PPI) network and hub genes were constructed and screened through the weighted gene co-expression analysis (WGCNA) algorithm and machine learning methods based on the two subgroups. Moreover, the immune cells infiltration level for each AS sample were estimated, and the relationship between immune cells, subgroups, and hub genes was further explored. These findings might improve our understanding of the molecular pathogenesis of AS and identify potential markers for the diagnosis of AS.

## Materials and Methods

### Data Collection

Three expression profile data sets including GSE20129, GSE43292, and GSE57691 were downloaded from the Gene Expression Omnibus database using the GEOquery R package (Davis and Meltzer, 2007). The GSE20129 dataset consisted of two independent sets of data generated using two different General Public License (GPL) platforms. The dataset with the largest number of samples (*N* = 119) generated using the GPL6104 platform (Illumina humanRef-8 v2.0 expression beadchip) was selected for further analysis. In addition, the GSE20129 samples were collected from the peripheral blood cell. The GSE43292 samples were originated from carotid atheroma plaque, and the GPL platform for GSE43292 was GPL6244 ([HuGene-1_0-st] Affymetrix Human Gene 1.0 ST Array [transcript (gene) version]), which includes 32 AS samples and 32 normal samples. The GSE57691 were derived from atherosclerotic vascular tissues, and the GPL platform for GSE57691 was GPL10558 (Illumina HumanHT-12 V4.0 expression beadchip), which contained nine AS samples and 10 normal samples (Supplementary Table 1).

### Batch Effect Removal

The sva R package was used to filter out any batch effect resulting from the combination of the two datasets (Leek et al., 2012). The expression values of the datasets (GSE20129 and GSE43292) were transformed using log2 before cross-platform normalization was carried out. We then used the comBat tool to eliminate the batch effect between the two data sets. Principal component analysis was applied to evaluate the performance of the comBat tools.

### Clustering of AS Samples

The non-negative matrix factorization (NMF) algorithm was used to carry out clustering analysis to identify potential clustering groups of the AS samples (Possemato et al., 2011). The cophenetic correlation coefficients were used to determine the optimal cluster number.

### Gene Set Variation Analysis

Gene set variation analysis (GSVA) is a non-parametric and unsupervised gene set enrichment method that evaluates the association between biological pathways and gene signatures based on expression profile data (Hänzelmann et al., 2013). The Kyoto Encyclopedia of Genes and Genomes (KEGG) pathway analysis data were retrieved from the c2.cp.kegg.v7.1.symbols.gmt file. Using GSEA analysis, the score for each AS samples on KEGG pathway was calculated. The pathways in which the gene signatures were enriched were identified using the “limma” R package with the cutoff fdr <0.05 and | log2FC| ≥ 0.2 (Ritchie et al., 2015; Yang et al., 2020).

### Weighted Gene Co-expression Network Analysis

Weighted gene co-expression analysis was conducted to identify potential modules that characterize the pathways or function of the subgroups on the basis of gene expression profile (Langfelder and Horvath, 2008). Since there was no significant difference in the expression levels of some genes, only genes with the highest 25% variance were used for WGCNA analysis. Pearson correlation analysis was carried out between gene pairs and the results used to construct the corresponding Pearson correlation matrix. The absolute values of the correlation co-efficient between the gene pairs were considered to be the co-expression similarity matrix. The power function formula, amn = | cmn| β (cmn represent the Pearson correlation between gene m and gene n; amn represent the adjacency between gene m and gene n), was used to build a weighted adjacency matrix. Moreover, we chose a β value to enhance the similarity matrix and achieve a scale free co-expression network (scale free R^2^ = 0.9). The adjacency matrix was further converted into a topological overlap matrix (TOM) and module dendrograms were constructed using average linkage hierarchical clustering methods. The gene in the min modules was at least 30. After merging the gene modules on the basis of similarity, we identified six valuable modules. The Pearson correlation coefficient was calculated between modules and subgroups using the cor function in the WGCNA R package.

### Enrichment of the Modules and Protein–Protein Network Construction

The “clusterProfiler” R packages were used to perform gene ontology (GO) and KEGG enrichment analysis for the genes contained in the modules (Van den Berg et al., 2009). Functions or pathways that were significantly enriched were identified based on the criterion: adjusted *P* < 0.05. The STRING database was used to construct the PPI network (Yang et al., 2020), while the cytoscape software was used to visualize the network. The key modules and genes were identified using the MCODE function within cytoscape with the criterion: score >2 (Szklarczyk et al., 2018).

### Identification of Key Genes

After selecting genes from MCODE module, we performed least absolute shrinkage and selection operator (LASSO) analysis with a turning/penalty parameter conducted using 10-fold cross validation to find the most relevant genes. LASSO is a dimension-reduction algorithm that has superiority in analyzing high-dimensional data when compared to regression analysis (Tibshirani, 1996; Bader and Hogue, 2003). Moreover, the support vector machine-recursive feature elimination (SVM-RFE) was also used to identify the key genes. SVM-RFE is a machine learning algorithm to identify the best variables by deleting SVM-generated eigenvectors (Huang et al., 2014). Finally, we incorporated the genes from LASSO analysis and SVM-RFE analysis to serve as the target genes.

### Evaluation of the Immune Cell Infiltration

The gene expression profiles of the AS samples were uploaded to CIBERSORTx online tools and the LM22 signature was used as the reference for 1,000 permutations (Ma and Li, 2018). The proportion of each immune cell was calculated through bulk-mode batch correction and absolute mode algorithm and an immune cells matrix was generated. Only samples with a *P* value <0.05 were selected for further analysis. The correlation coefficient between immune cells was calculated using the “corrplot” R package, while the Wilcoxon test was used to estimate the difference between subgroups. Moreover, the receiver operating characteristic (ROC) curve was applied to evaluate the accuracy of the key genes.

## Results

### Data Preprocessing

**FIGURE 1:**
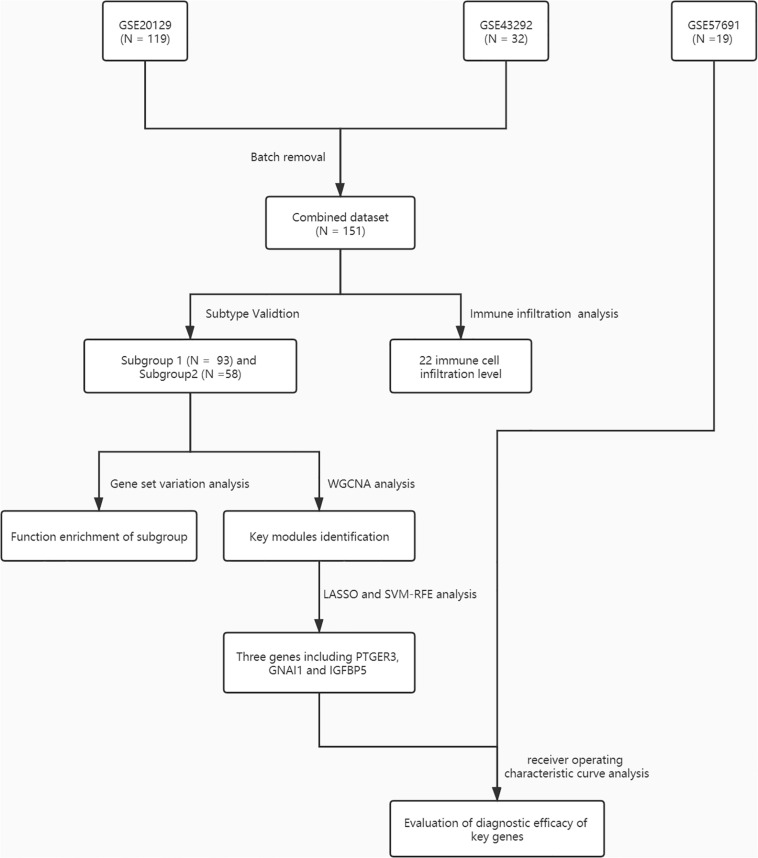
A flowchart for the analysis procedure to identify potential molecular subtypes and key genes in this study.

**FIGURE 2:**
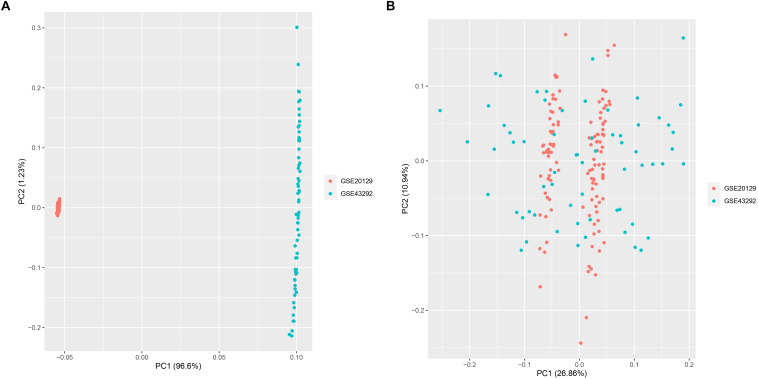
Principal component analysis (PCA) for the two datasets (GSE20129 and GSE43292) before **(A)** and after merge **(B)**.

A total of 151 AS samples and 32 normal samples from GSE20129 and GSE43292 were used as the training dataset, and GSE57691 (*N* = 19) data set served as the external validation data set. The detailed flowchart was presented in Figure 1. We then used the combat method to filter batch effects after combining gene expression data from the two training datasets and remained with data for 13,460 genes. We then conducted principle component analysis (PCA) on the two data sets to establish the relationship between the two training datasets. As shown in Figure 2A, samples from the two independent data sets formed different clusters before batch effect removal, but clustered together after batch effect removal (Figure 2B). This indicated that cross-platform normalization had successfully removed the batch effect.

### NMF Clustering of AS Samples and GSVA Enrichment Analysis

**FIGURE 3:**
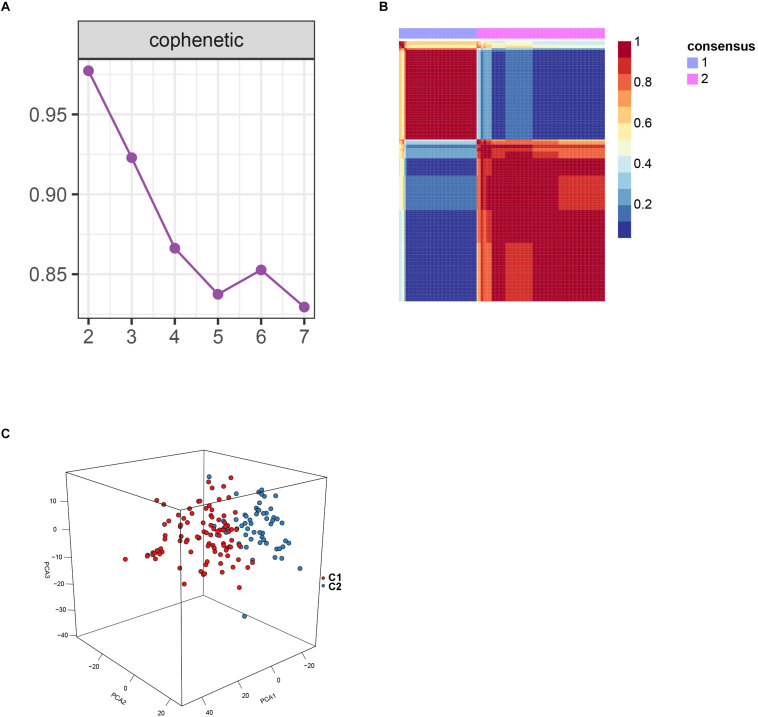
Non-negative matrix factorization analysis for the merge dataset. **(A)** The cophenetic correlation coefficient was calculated for *k* = 2–7. **(B)** Non-negative matrix heatmap was plotted when *k* = 2. **(C)** Principal component analysis (PCA) supported the stratification when *k* = 2.

**FIGURE 4:**
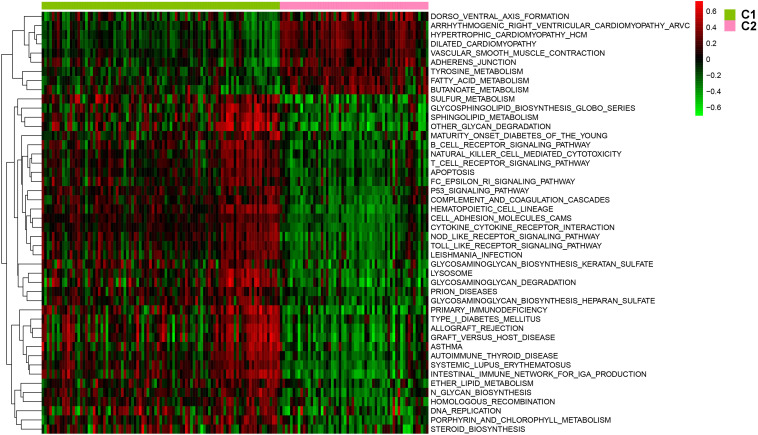
The involved pathway between two distinct subtypes were identified via the Gene Set Variation Analysis (GSVA) analysis.

After batch effect was filtered, the gene expression profiles of 151 AS samples were used to carry out NMF cluster analysis. To identify the potential AS subgroups, we selected the top 1,000 variance genes for the clustering analysis. The cophenetic correlation coefficients was used to determine the optimal k number, with the results showing that *k* = 2 was the optimal subgroup number (Figures 3A,B). Results of PCA analysis showed that there was significant difference between the two subgroups (Figure 3C). Consequently, we divided the AS samples into two subgroups, subgroup 1 (*N* = 93) and subgroup 2 (*N* = 58). We further used the GSVA enrichment analysis to compare the pathways enriched in the two groups. As shown in Figure 4, the pathways associated with subgroup 1 include p53 signaling pathway, apoptosis, T cell receptor signaling pathway, B cell receptor signaling pathway, NOD_LIKE receptor signaling pathway, and TOLL_LIKE receptor signaling pathway. On the other hand, the pathways enriched in subgroup 2 include fatty acid metabolism, adherens junction, and tyrosine metabolism.

### Gene Co-expression Network Construction and Module Enrichment

**FIGURE 5:**
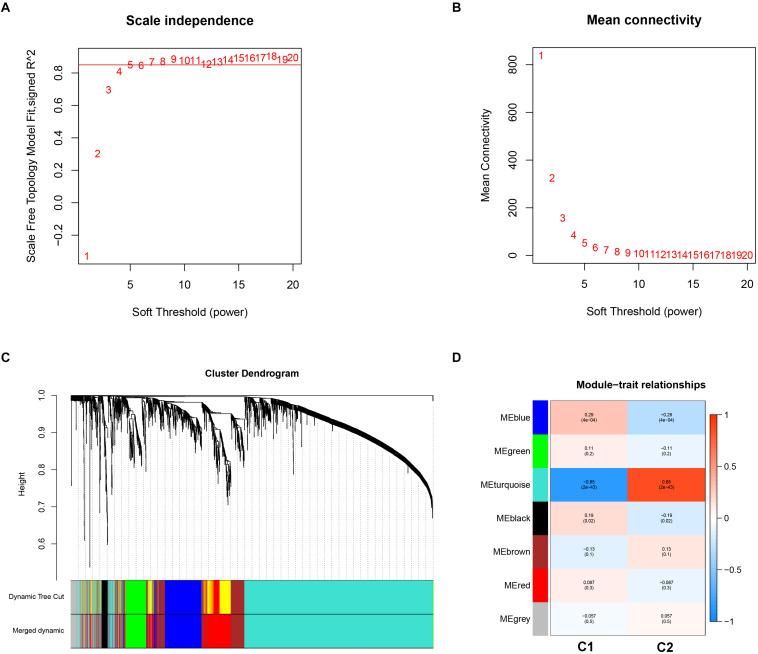
Key modules identification of the two subgroups using weighted correlation network analysis. **(A)** Scale-free fitting index analysis for different soft-thresholding forces (β). **(B)** Mean connectivity for different soft-thresholding powers. **(C)** Clustering dendrograms for the top 25% variance genes on the basis of dissimilarity topological overlap and module colors. **(D)** Module-trait heatmap was constructed by using the correlation coefficient.

Weighted gene co-expression analysis analysis was performed to construct a co-expression network to identify potential modules most relevant to the AS subgroups. First, in order to obtain good quality results, genes with the highest 25% of variance in their gene expression profiles were used to carry out WGCNA analysis. As a result, clustering analysis was carried out on 3,365 genes with 151 AS samples using the average linkage method and Pearson’s correlation method (Supplementary Figure 1). In the study, the soft threshold power value of β = 5 soft power was selected to obtain a scale-free co-expression network (scale-free R^2^ = 0.85). From this analysis six modules were used for further analysis (Figures 5A–C).

**FIGURE 6:**
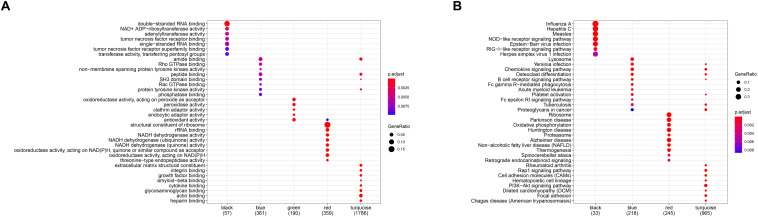
Gene ontology (GO) **(A)** and Kyoto Encyclopedia of Genes and Genomes (KEGG) **(B)** enrichment analysis for all gene modules.

In order to investigate the association between modules and the AS subgroups, the module significance (MS) was employed as the gene expression level of the corresponding module to calculate the Pearson correlation coefficient between the module and the subgroups. As shown in Figure 5D, the turquoise module had the highest correlation with subgroup 1 (cor = -0.85) and subgroup 2 (cor = 0.85) and was therefore selected for subsequent analysis. In addition, to systemically investigate the function and pathway of each module, we further performed GO and KEGG pathway enrichment analysis. As shown in Figure 6A, the GO enrichment terms were different among the modules suggesting that these modules play different roles in the subgroups. However, the results of KEGG pathway analysis revealed that similar pathways were enriched in the blue and turquoise module, indicating that the two modules were similar (Figure 6B).

### Protein–Protein Network Construction and Key Modules Selecting

**FIGURE 7:**
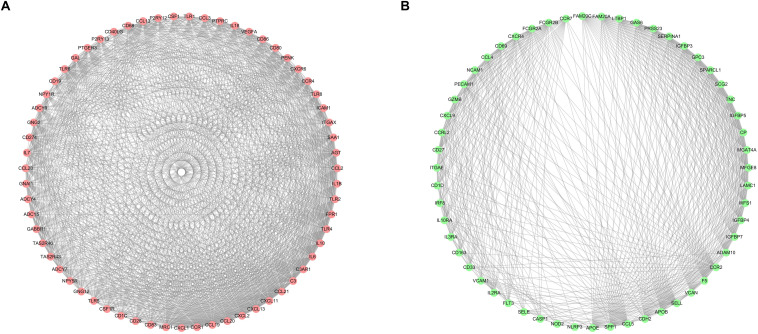
Identification of the top two sub networks by using the MCODE algorithm. **(A)** The first sub network. **(B)** The second sub network.

**FIGURE 8:**
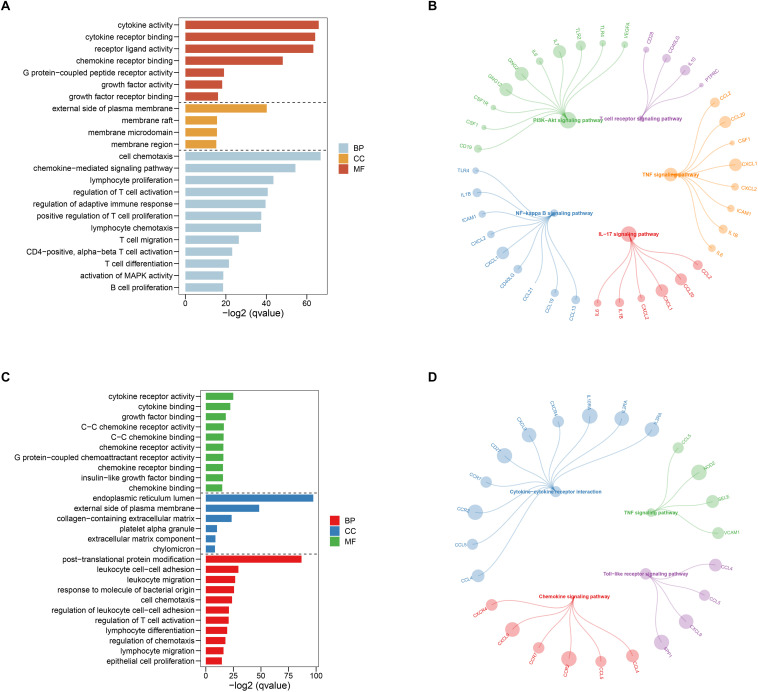
Gene ontology (GO) and Kyoto Encyclopedia of Genes and Genomes (KEGG) enrichment analysis for the first **(A,B)** and second **(C,D)** sub networks, respectively.

After selecting the turquoise module from the WGCNA analysis, we further investigated the role of the genes in the module. We then used the genes to build the PPI network based on STRING database and used the cytoscape software to visualize the network, in which a total of 23,023 edges and 1,835 nodes were incorporated. The network was processed using the MCODE module to identify possible key modules and the top two important modules acquired (Figure 7). Results of GO and KEGG analysis showed that the genes in the key module 1 and key module 2 were significantly enriched in the immune-related pathways and function (Figure 8).

### Screening and Verification of Potential Diagnostic Markers

**FIGURE 9:**
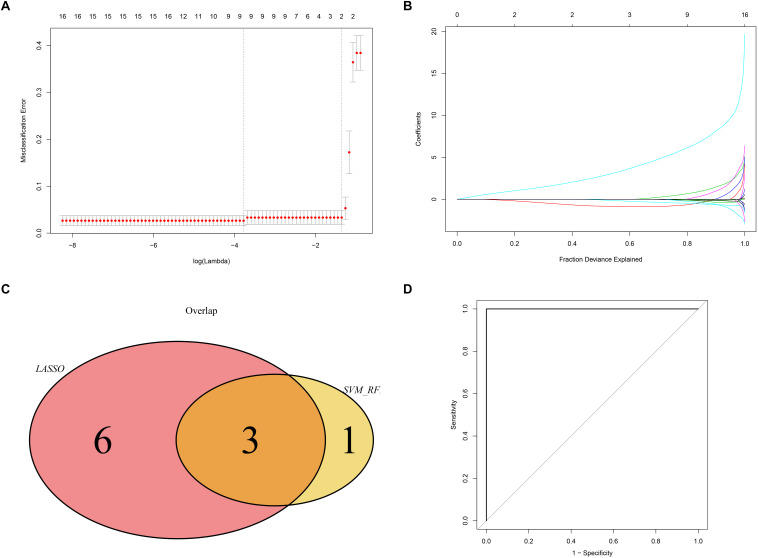
Screening and validation of biomarkers. **(A)** L1-penalty of Least absolute shrinkage and selection operator (LASSO) regression. The dotted vertical lines denote the optimal value: 9. **(B)** LASSO coefficient values of the sub networks genes. **(C)** The Venn graph shows the interaction between LASSO and SVM-RFE algorithm. **(D)** Validation of the diagnostic value in the external dataset by using receiver operating characteristic curve (ROC) analysis.

We used the LASSO and SVM-RFE algorithms to identify robust genes from the key modules. The algorithm identified nine genes (Figures 9A,B), while the SVM-RFE algorithm identified 4 genes (Supplementary Figure 2). Three of the genes identified by the two algorithms overlapped including *PTGER3*, *GNAI1*, and *IGFBP5*, suggesting that these were the robust genes for AS (Figure 9C). The diagnostic efficacy of *PTGER3*, *GNAI1*, and *IGFBP5* was determined using a validation dataset. When *PTGER3*, *GNAI1*, and *IGFBP5* were fitted into one variable, the diagnostic efficiency reached a higher level in the validation set (AUC = 1) (Figure 9D), indicating that *PTGER3*, *GNAI1*, and *IGFBP5* had high diagnostic value for the AS.

### Immune Cell Infiltration Analysis

**FIGURE 10:**
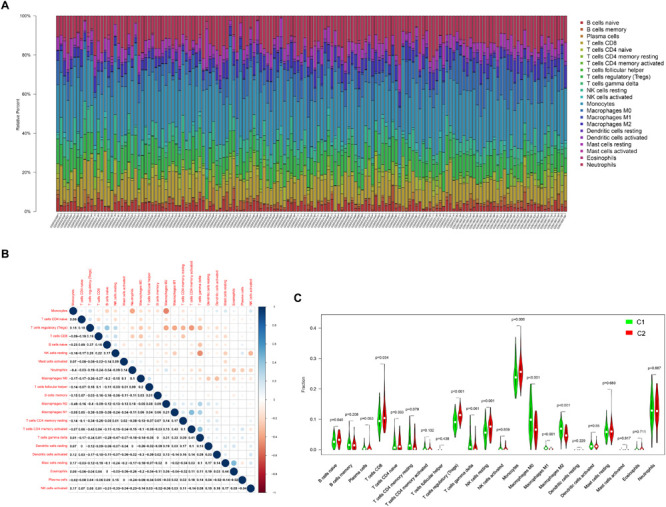
The landscape of the level of infiltration of AS by 22 immune cells. **(A)** Barplot of the 22 immune cells infiltration level. **(B)** Correlation analysis of the 22 immune cells. **(C)** The comparisons of the 22 immune cells infiltration level between subtype C1 and C2.

**FIGURE 11:**
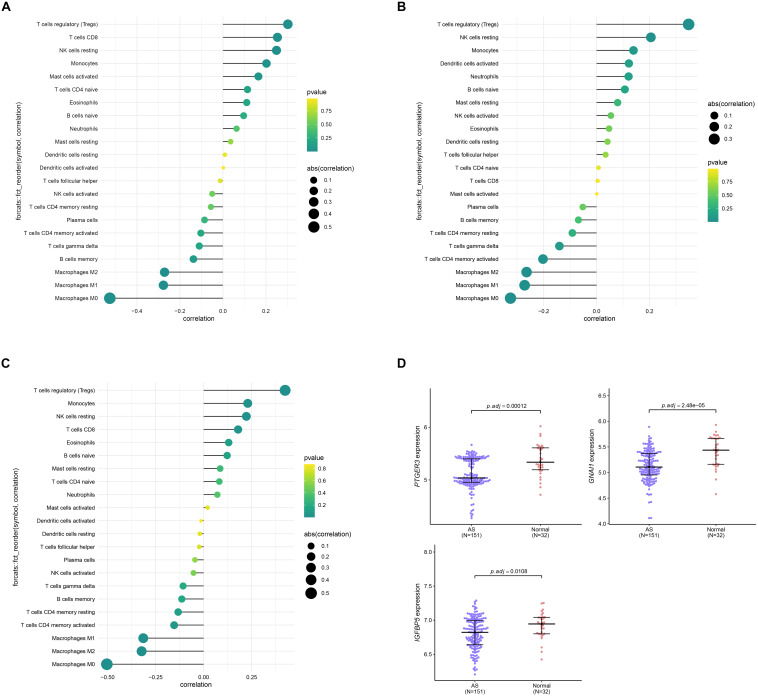
Correlation analysis between the three genes *PTGER3* **(A)**, *GNAI1* **(B)**, *IGFBP5* **(C)**, and 22 immune cells infiltration level. **(D)** The expression level of three genes between normal and AS tissue.

Since results of GO and KEGG enrichment analysis showed that the genes were enriched in immune related pathways, we used the CIBERSORTx online tool to determine the level of immune cell infiltration. Samples were considered to have immune cells infiltration if the *P* value < 0.05. The distribution of immune cells was showed in a barplot (Figure 10A), and the correlation between immune cells was calculated using the corrplot R package (Figure 10B). Moreover, we further explored the association between immune cells and the AS subgroups. The results of this analysis showed that there was a significant difference in the infiltration level of na ve B cells, CD8 T cells, Tregs, resting NK cells, Monocytes, Macrophages M0, Macrophages M1, and Macrophages M2 between the two subgroups (Figure 10C). In addition, we found that the three key genes were positively correlated with Tregs and negatively correlated with Macrophages M0 (Figure 11A–C). Interestingly, the expression levels of the three genes were significantly high in the normal tissue compared to AS tissue (Figure 11D).

## Discussion

Recently, AS has been associated with high morbidity and mortality (Ma and Li, 2018). AS is a heterogeneous disease, and its pathogenesis is complex and poorly understood. Therefore, there is a compelling need to identify biomarkers that might be useful for diagnosing AS. Liu et al., using the GSE20129 dataset, revealed five genes including *APH1B*, *JAM3*, *FBLN2*, *CSAD*, and *PSTPIP2* play an essential role in the progression of AS and may be potential biomarkers for diagnosis of AS (Liu et al., 2016). Huang et al. discovered three differentially expressed genes (*KDELR3*, *CD55*, and *DYNC2H1*) can serve as diagnostic and therapeutic targets in AS macrophages through microarray analysis (Huang et al., 2019). Wang et al. (2019) constructed a miRNA-TF-gene network through microarray data analysis in the AS macrophages. However, these studies only concentrated on the characterization of potential biomarkers, and the samples size of the dataset is small, which leads to low accuracy. A previous review reported that a small sample size could exert an impact on biomarker identification due to the heterogeneity of AS, especially in the mRNA level (Chen and Stewart, 2016). Considering that, we incorporated multiple data sets to identify the robust subgroups of AS to better understand the underlying molecular pathogenesis of AS. Using the NMF algorithm, we successfully divided the AS samples into two subgroups. Results of PCA analysis showed that our transcriptome classification was robust. Moreover, GSVA enrichment analysis showed that subgroup 1 and subgroup 2 were associated with different pathways. Subgroup 1 was enriched in the immune-related pathways, while subgroup 2 was enriched in the metabolism pathways. Taken together, the transcriptome classification of AS was closely associated with specific pathways, which may have important clinical implications for the treatment of AS.

Weighted gene coexpression network analysis is a systematic biological method used to identify key gene modules associated with phenotypic traits (Botía et al., 2017). Compared to thousands of genes focus on differential expression, WGCNA can exploit thousands of most variable genes or all genes to explore the relationship between gene module and clinical traits (Wan et al., 2017; Tang et al., 2019). It can provide a specific measure for clinical prediction of AS diagnosis. Here, the association of gene modules and subgroups was investigated using WGCNA analysis. We identified the turquoise module is an interest module that correlated with the molecular subgroups. Further analysis using the LASSO and SVM-RFE algorithms identified three robust key genes including *PTGER3*, *GNAI1*, and *IGFBP5* from the module. Among these genes, *PTGER3* is usually involved in TLR4/PTGS2 signaling and has been identified as an potential biomarker in atherosclerotic plaque (Ferronato et al., 2018). *IGFBP5* (insulin like growth factor binding protein 5) is epigenetically silenced by H3K27me3 in advanced atherosclerotic plaques, suggesting that targeting the H3K27me3/IGFBP5 pathway may provide novel therapeutics for atherosclerosis (Xu et al., 2018). *GNAI1* (G Protein Subunit Alpha I1) has been identified as a potential biomarker in multiple tumors, such as ovarian cancer, colorectal cancer, and renal cell carcinoma, but its role in AS has not yet been reported (Liang et al., 2016; Zhan et al., 2018). These results indicated that the three genes play a crucial role in the development of AS. In addition, the three genes *PTGER3*, *GNAI1*, and *IGFBP5* showed a high performance in the validation data set, indicating that they have potential as diagnostic markers in AS. However, *in vitro* experiments or *in vivo* experiments are required to validate these findings.

Since the subgroups were enriched in immune related pathways, we further investigated the association between immune cells and the AS subgroups. We discovered that the naïve B cells, T CD8 cells, Tregs, resting NK cells, and Monocytes are highly expressed in subgroup 2 compared to group 1, while Macrophages M0, Macrophages M1, and Macrophages M2 had low expression levels in subgroup 2. Previously studies have reported that macrophages play a central role in the development of atherosclerosis. In each vascular bed, macrophages contribute to the maintenance of the local inflammatory response, propagate plaque development, and promote thrombosis (Moore and Tabas, 2011; Moore et al., 2013). Thus, we speculate that subgroup 1 is more prone to developing advanced AS compared to subgroup 2, and further studies should be implemented to validate the result.

Despite the molecular subtypes and key genes associated with AS that have been identified in this study, several limitations should be elucidated. First, AS may have distinct pathologies in gender and age, which exert an impact on our result. Second, due to the different GPL version platforms, tissue source, and experimental treatment of these datasets, the batch of effect could not be completely removed. Third, the sample size for the current study is relative small and may affect the accuracy of the result. Finally, there was no experimental validation in this study, and further validation should be carried out to validate these findings and speculations.

In summary, we identified two novel subgroups of AS, which provide novel insight into cellular and molecular characteristics of AS. Moreover, we identified three key genes including *PTGER3*, *GNAI1*, and *IGFBP5* as potential markers for diagnosis and targeted therapy of AS.

## Data Availability Statement

Publicly available datasets were analyzed in this study. The datasets generated for this study can be found in the https://www.ncbi.nlm.nih.gov/geo/.

## Author Contributions

YY designed the study and collected the dataset. YC and YZ analyzed the dataset. YY and YC wrote the manuscript. XY and ZX revised the manuscript. All authors contributed to the article and approved the submitted version.

## Conflict of Interest

The authors declare that the research was conducted in the absence of any commercial or financial relationships that could be construed as a potential conflict of interest.

## Supplementary Material

Supplementary Figure 1 Dendrogram of clustering of all AS samples in the merge dataset.

Supplementary Figure 2 Identification of key genes using the Support vector machine-recursive feature elimination (SVM-RFE) algorithm.

Supplementary Table 1 The information (GPL version, samples) of the three dataset.

## Abbreviations

ASCVD: atherosclerotic cardiovascular disease
AS: Atherosclerosis
CRP: C-reactive protein
GO: gene ontology
GPL: General Public License
GSVA: gene set variation analysis
KEGG: Kyoto Encyclopedia of Genes andGenomes
LASSO: least absolute shrinkage and selection operator
MS: module significance
NMF: non-negative matrix factorization
PCA: principle component analysis
PPI: protein-protein network
ROC: receiver operating characteristic
SVM-RFE: support vector machine-recursive feature elimination
TOM: topological overlap matrix
Tregs: T regulatory cells
WGCNA: weighted gene co-expression analysis.

## References

[B1] Bader G. Hogue C. (2003). An automated method for finding molecular complexes in large protein interaction networks. *BMC Bioinform.* 4:2. 10.1186/1471-2105-4-2 12525261 PMC149346

[B2] Botía J. Vandrovcova J. Forabosco P. Guelfi S. D’Sa K. Hardy J. (2017). An additional k-means clustering step improves the biological features of WGCNA gene co-expression networks. *BMC Syst. Biol.* 11:47. 10.1186/s12918-017-0420-6 28403906 PMC5389000

[B3] Chen H.-H. Stewart A. (2016). Transcriptomic signature of atherosclerosis in the peripheral blood: fact or fiction? *Curr. Atherosclerosis Rep.* 18:77. 10.1007/s11883-016-0634-x 27815828

[B4] Davis S. Meltzer P. (2007). GEOquery: a bridge between the gene expression omnibus (GEO) and BioConductor. *Bioinformatics* 23 1846–1847. 10.1093/bioinformatics/btm254 17496320

[B5] Ferronato S. Scuro A. Fochi S. Orlandi E. Gomez-Lira M. Olivato S. (2018). Expression of TLR4-PTGE2 signaling genes in atherosclerotic carotid plaques and peripheral blood. *Mol. Biol. Rep.* 46 1317–1321. 10.1007/s11033-018-4478-z 30421129

[B6] Hänzelmann S. Castelo R. Guinney J. G. S. V. A. (2013). Gene set variation analysis for microarray and RNA-Seq data. *BMC Bioinform.* 14:7. 10.1186/1471-2105-14-7 23323831 PMC3618321

[B7] Huang H.-M. Jiang X. Hao M.-L. Shan M.-J. Qiu Y. Hu G.-F. (2019). Identification of biomarkers in macrophages of atherosclerosis by microarray analysis. *Lipids Health Dis.* 18:107. 10.1186/s12944-019-1056-x 31043156 PMC6495566

[B8] Huang M. L. Hung Y.-H. Lee W. Li R. Jiang B.-R. (2014). SVM-RFE Based feature selection and taguchi parameters optimization for multiclass SVM classifier. *TheScientificWorldJournal* 2014:795624. 10.1155/2014/795624 25295306 PMC4175386

[B9] Jeong I.-K. (2010). Molecular biology of atherosclerosis. *Endocrinol. Metab.* 25 166–170. 10.3803/EnM.2010.25.3.166

[B10] Kampoli A.-M. Tousoulis D. Antoniades C. Siasos G. Stefanadis C. (2009). Biomarkers of premature atherosclerosis. *Trends Mol. Med.* 15 323–332. 10.1016/j.molmed.2009.06.001 19577961

[B11] Kelly P. J. Murphy S. Coveney S. Purroy F. Lemmens R. Tsivgoulis G. (2018). Anti-inflammatory approaches to ischaemic stroke prevention. *J. Neurol. Neurosurg. Psychiatry* 89 211–218. 10.1136/jnnp-2016-314817 28935831

[B12] Kong Q. X. Xia M. Liang R. Q. Li L. Hu J. (2014). Increased serum visfatin as a risk factor for atherosclerosis in patients with ischaemic cerebrovascular disease. *Singapore Med. J.* 55 383–387. 10.11622/smedj.2014091 25091888 PMC4291965

[B13] Langfelder P. Horvath S. (2008). WGCNA: an R package for weighted correlation network analysis. *BMC Bioinformatics* 9:559. 10.1186/1471-2105-9-559 19114008 PMC2631488

[B14] Leek J. Johnson W. Parker H. Jaffe A. Storey J. (2012). The SVA package for removing batch effects and other unwanted variation in high-throughput experiments. *Bioinformatics* 28 882–883. 10.1093/bioinformatics/bts034 22257669 PMC3307112

[B15] Liang B. Li C. Zhao J. (2016). Identification of key pathways and genes in colorectal cancer using bioinformatics analysis. *Med. Oncol.* 33:111. 10.1007/s12032-016-0829-6 27581154

[B16] Liu L. Liu Y. Liu C. Zhang Z. Du Y. Zhao H. (2016). Analysis of gene expression profile identifies potential biomarkers for atherosclerosis. *Mol. Med. Rep.* 14 3052–3058. 10.3892/mmr.2016.5650 27573188 PMC5042771

[B17] Ma J. Li H. (2018). The role of gut microbiota in atherosclerosis and hypertension. *Front. Pharmacol.* 9:1082. 10.3389/fphar.2018.01082 30319417 PMC6167910

[B18] Montagnana M. Lippi G. Salvagno G. Franchini M. Targher G. Guidi G. (2008). Role of biochemical risk factors and markers for the risk of atherosclerosis. *Minerva Med.* 99 215–222. 18595637

[B19] Moore K. Sheedy F. Fisher E. (2013). Macrophages in atherosclerosis: a dynamic balance. *Nat. Rev. Immunol.* 13 709–721. 10.1038/nri3520 23995626 PMC4357520

[B20] Moore K. Tabas I. (2011). Macrophages in the pathogenesis of atherosclerosis. *Cell* 145 341–355. 10.1016/j.cell.2011.04.005 21529710 PMC3111065

[B21] Niccoli G. Montone R. A. Sabato V. Crea F. (2018). Role of allergic inflammatory cells in coronary artery disease. *Circulation* 138 1736–1748. 10.1161/circulationaha.118.035400 30354461

[B22] Possemato R. Marks K. Shaul Y. Pacold M. Kim D. Birsoy K. (2011). Functional genomics reveals serine synthesis is essential in PHGDH-amplified breast cancer. *Nature* 476 346–350. 10.1038/nature10350 21760589 PMC3353325

[B23] Revkin J. Shear C. Pouleur H. Ryder S. Orloff D. (2007). Biomarkers in the prevention and treatment of atherosclerosis: need, validation, and future. *Pharmacol. Rev.* 59 40–53. 10.1124/pr.59.1.1 17329547

[B24] Ritchie M. Phipson B. Wu D. Hu Y. Law C. Shi W. (2015). LIMMA powers differential expression analyses for RNA-sequencing and microarray studies. *Nucleic Acids Res.* 43:e47. 10.1093/nar/gkv007 25605792 PMC4402510

[B25] Stefanadis C. Antoniou C. K. Tsiachris D. Pietri P. Disease C. (2017). Coronary atherosclerotic vulnerable plaque: current perspectives. *J. Am. Heart Assoc.* 6:e005543. 10.1161/JAHA.117.005543 PMC5524044 28314799

[B26] Szklarczyk D. Gable A. Lyon D. Junge A. Wyder S. Huerta-Cepas J. (2018). STRING v11: protein-protein association networks with increased coverage, supporting functional discovery in genome-wide experimental datasets. *Nucleic Acids Res.* 47 D607–D613. 10.1093/nar/gky1131 30476243 PMC6323986

[B27] Tang X. Huang X. Wang D. Yan R. Lu F. Cheng C. (2019). Identifying gene modules of thyroid cancer associated with pathological stage by weighted gene co-expression network analysis. *Gene* 704 142–148. 10.1016/j.gene.2019.04.017 30965127

[B28] Tern P. J. W. Kujawiak I. Saha P. Berrett T. B. Chowdhury M. M. Coughlin P. A. (2018). Site and burden of lower limb atherosclerosis predicts long-term mortality in a cohort of patients with peripheral arterial disease. *Eur. J. Vasc. Endovasc. Surg.* 56 849–856. 10.1016/j.ejvs.2018.07.020 30287208

[B29] Tibshirani R. (1996). Regression shrinkage and selection via the lasso. *J. R. Stat. Soc. Ser. B* 58 267–288. 10.1111/j.2517-6161.1996.tb02080.x

[B30] Van den Berg B. Thanthiriwatte C. Manda P. Bridges S. (2009). Comparing gene annotation enrichment tools for functional modeling of agricultural microarray data. *BMC Bioinform.* 10(Suppl. 11):S9. 10.1186/1471-2105-10-S11-S9 19811693 PMC3226198

[B31] Wan Q. Tang J. Han Y. Wang D. (2017). Co-expression modules construction by WGCNA and identify potential prognostic markers of uveal melanoma. *Exp. Eye Res.* 166 13–20. 10.1016/j.exer.2017.10.007 29031853

[B32] Wang W. Zhang K. Zhang H. Li M. Zhao Y. Wang B. (2019). Underlying genes involved in atherosclerotic macrophages: insights from microarray data mining. *Med. Sci. Monit.* 25 9949–9962. 10.12659/MSM.917068 31875420 PMC6944040

[B33] Xu S. Xu Y. Yin M. Zhang S. Liu P. Koroleva M. (2018). Flow-dependent epigenetic regulation of IGFBP5 expression by H3K27me3 contributes to endothelial anti-inflammatory effects. *Theranostics* 8 3007–3021. 10.7150/thno.21966 29896299 PMC5996356

[B34] Yang C. Huang X. Liu Z. Qin W. Wang C. (2020). Metabolism-associated molecular classification of hepatocellular carcinoma. *Mol. Oncol.* 14 896–913. 10.1002/1878-0261.12639 31955511 PMC7138397

[B35] Zhan S. J. Liu B. Linghu H. (2018). Identifying genes as potential prognostic indicators in patients with serous ovarian cancer resistant to carboplatin using integrated bioinformatics analysis. *Oncol. Rep.* 39 2653–2663. 10.3892/or.2018.6383 29693178 PMC5983937

