# Supplementary figures and images for: Identification of Molecular Subtypes and Key Genes of Atherosclerosis Through Gene Expression Profiles
Source: Front Mol Biosci. 2021 Apr 28;8:628546. doi: 10.3389/fmolb.2021.628546 (PMC8113832; doi:10.3389/fmolb.2021.628546)

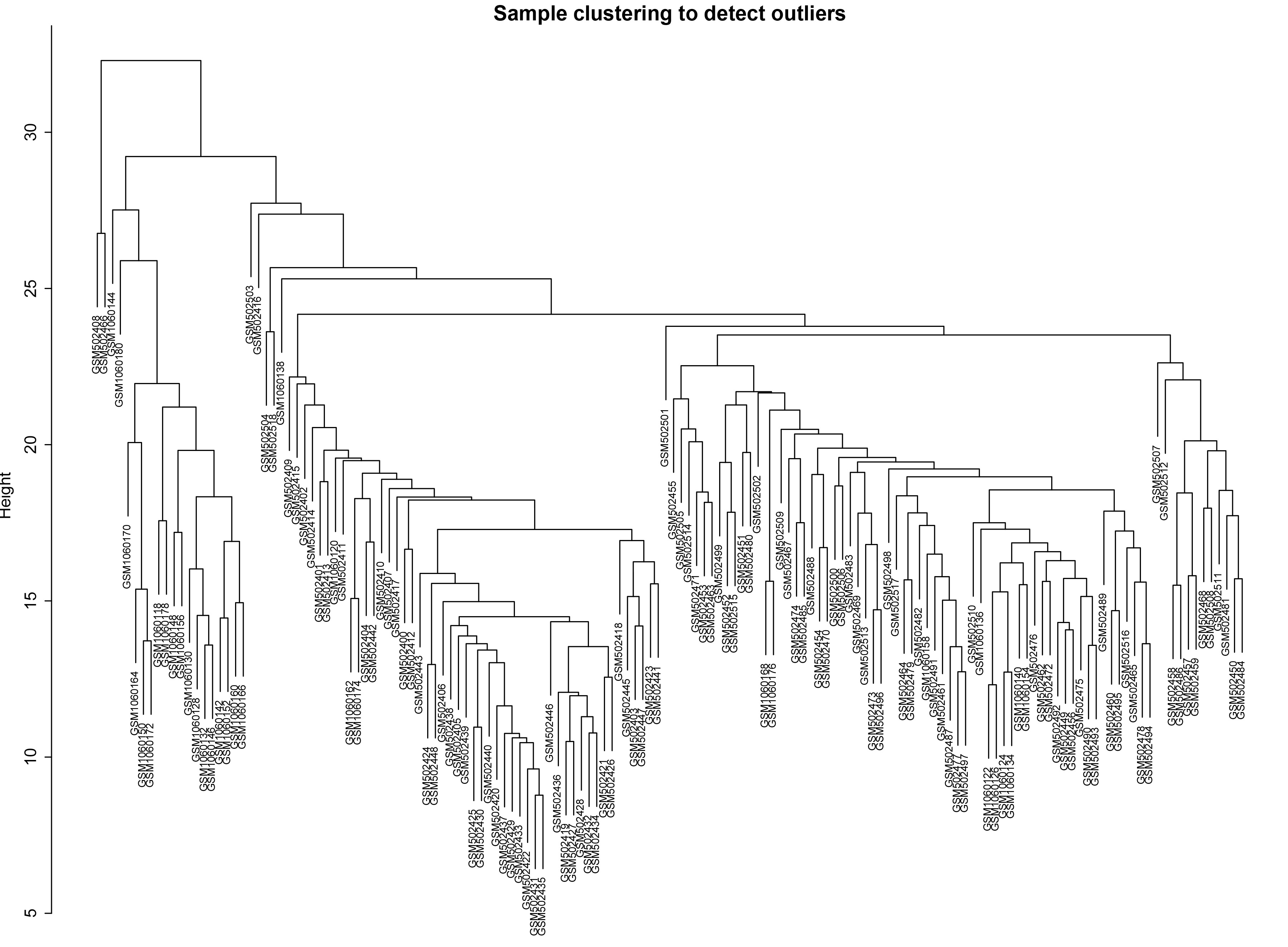

Supplement: Supplementary Figure 1 — Dendrogram of clustering of all AS samples in the merge dataset. [file Image_1.JPEG]

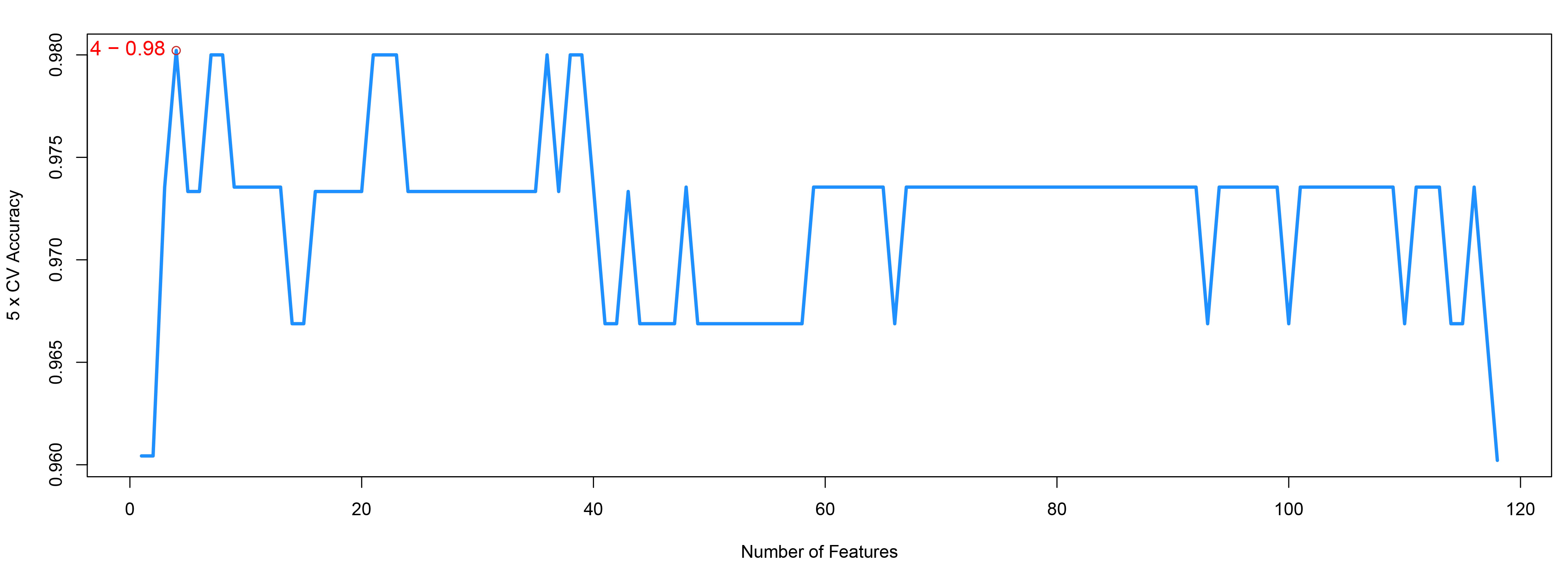

Supplement: Supplementary Figure 2 — Identification of key genes using the Support vector machine-recursive feature elimination (SVM-RFE) algorithm. [file Image_2.JPEG]
