# Supplementary material for: Identification of Molecular Subtypes and Key Genes of Atherosclerosis Through Gene Expression Profiles
Source: Front Mol Biosci. 2021 Apr 28;8:628546. doi: 10.3389/fmolb.2021.628546 (PMC8113832; doi:10.3389/fmolb.2021.628546)
Supplement: Supplementary Table 1 — The information (GPL version, samples) of the three dataset. [file Table_1.DOC]

| Dataset | Samples | GPL version |
| --- | --- | --- |
| GSE20129 | AS: 119 | GPL6104 |
| GSE43292 | AS: 32; Normal: 32 | GPL6244 |
| GSE57691 | AS: 9; Normal: 10 | GPL10558 |
